# Supplementary material for: High prevalence of active trachoma and associated factors among school-aged children in Southwest Ethiopia
Source: PLoS Negl Trop Dis. 2023 Dec 15;17(12):e0011846. doi: 10.1371/journal.pntd.0011846 (PMC10756553; doi:10.1371/journal.pntd.0011846)
Supplement: S1 File — (DOCX) [file pntd.0011846.s001.docx]

|  | Observe the availability of latrine in the HH | Yes (1) No (2) |
| --- | --- | --- |
|  | Type of latrine | ----------------- |
|  | Estimated distance of latrine from living house | ----------------- |
|  | Are flies observed around the toilet or in the compound? | Yes (1) No (2) |
|  | Separate room for cattle and cooking, | Yes (1) No (2) |
|  | presence of window in the cooking room | Yes (1) No (2) |
|  | Availability, liquid waste disposal | Yes (1) No (2) |
|  | Presence of hand washing facility after toilet | Yes (1) No (2) |
|  | Observed the presence of faeces in the compound | Yes (1) No (2) |
|  | Cleanliness of child face | Yes (1) No (2) |
|  | Cleanliness of fingers, | Yes (1) No (2) |
|  | Ocular discharge | Yes (1) No (2) |
|  | Nasal discharge | Yes (1) No (2) |
|  | Flies on Eye | Yes (1) No (2) |
|  | Flies on Face | Yes (1) No (2) |

**Observation cheek list**
