# Supplementary material for: High prevalence of active trachoma and associated factors among school-aged children in Southwest Ethiopia
Source: PLoS Negl Trop Dis. 2023 Dec 15;17(12):e0011846. doi: 10.1371/journal.pntd.0011846 (PMC10756553; doi:10.1371/journal.pntd.0011846)
Supplement: S2 File — (DOCX) [file pntd.0011846.s002.docx]

## **English version Questionnaires**

**INSTRUCTION**: The questionnaire has 6 parts and it will take about **30-45 minutes to complete the interview.**

Thank you very much for your patience.

Kebeles code ___________

Name of the data collector ________Date of interview __________Signature___________

Name of the supervisor ________ Date of supervision _________Signature___________

**Part I. Socio-demographic variables of the HH head and the child.**

| No | Questions | Response | | |
| --- | --- | --- | --- | --- |
| 101 | What is your age? | ________________ | | |
| 102 | What is your gender? | **1**.Male  2. Female | | |
| 103 | What is your religion? | 1. Orthodox 2. Muslim | | |
| 104 | What is your Marital status? | 1.Currently-married  2.Currently-not married | | |
| 105 | What is your educational status | 1. Unable to read& write 2. Abel to read & write only 3. Elementary school 4. Secondary school 5. Diploma & above | | |
| 106 | What is your or the HH head occupation**?** | 1. Governmental Employee 2. Marchant 3. Farmer or Housewife 4. Daily labourer | | |
| 107 | What is the place of residence | 1. Urban 2. Rural | | |
| 108 | What is the total Number of Family member living in this HH? | | | __________ |
| 109 | What is the total number children aged 1 to 9 years in this HH? | | | __________ |
| 101 | What is the Age of the selected child? | |  | |
| 111 | What is the Sex of the selected child? | | **1**.Male 2. Female | |
| 112 | Does the child was enrolled in school | | **1**.Yes 2. No | |
| 113 | What is the child grade level ? | | **______________** | |

**Part II. Water Sanitation and Hygiene related factors**

| 201 | What is the source of drinking water for the HH? | 1. Piped water into dwelling 2. Piped water to yard/plot 3. Public tap/standpipe 4. Protected dug well 5. Unprotected dug well 6. Protected spring 7. Unprotected spring 8. Rainwater collection 9. Surface water (river, dam, lake) 10. Pond, stream, canal, irrigation Channels) | |
| --- | --- | --- | --- |
| 202 | What is the main source of water used by your household for other purposes, such as cooking and hand washing? | 1. Piped water into dwelling 2. Piped water to yard/plot 3. Public tap/standpipe 4. Protected dug well 5. Unprotected dug well 6. Protected spring 7. Unprotected spring 8. Rainwater collection 9. Surface water (river, dam, lake) 10. Pond, stream, canal, irrigation Channels) | |
| 204 | How long does it take to go there, get water, and come back? | | 1.Water source found on premises  2. if not No. Of minutes or hour it takes to reach _______________ |
| 207 | What kind of toilet facility do members of your household usually use? If “flush” or “pour flush” probe: Where does it flush to? | 1. Flush/pour flush to 2. Ventilated improved pit latrine (VIP) 3. Pit latrine with slab 4. Pit latrine without slab/open pit 5. No facilities or bush or fi eld | |
| 208 | Who can use the toilet facilities? | 1. All family member 2. Only adult family member | |

**Part III. Child behavioral factor**

| 301 | Does the child face his/her face every morning? | Yes (1) No (2) |
| --- | --- | --- |
| 302 | How many times the child face washed per day | __________ |
| 303 | Does the child use soap while washing his face | Yes (1) No (2) |
| 304 | Does the child wash his hand after toilet? | Yes (1) No (2) |
| 305 | Does the child use soap to wash his hand | Yes (1) No (2) |
| 306 | Does the child have habit of sharing towel | Yes (1) No (2) |

**Part IV. House hold Asset**

|  | Ox | | Yes (1) No (2) |
| --- | --- | --- | --- |
|  | Cow | | Yes (1) No (2) |
|  | Calf | | Yes (1) No (2) |
|  | Sheep | | Yes (1) No (2) |
|  | Goat | | Yes (1) No (2) |
|  | Horse | | Yes (1) No (2) |
|  | Donkey | | Yes (1) No (2) |
|  | Cock and Hen | | Yes (1) No (2) |
|  | Television | | Yes (1) No (2) |
|  | Radio | | Yes (1) No (2) |
|  | Electricity | | Yes (1) No (2) |
|  | Refrigerator | | Yes (1) No (2) |
|  | Conventional telephone | | Yes (1) No (2) |
|  | Mobile phone | | Yes (1) No (2) |
|  | Motorcycle | | Yes (1) No (2) |
|  | Cycle | | Yes (1) No (2) |
|  | Cart | | Yes (1) No (2) |
|  | Gold, money | | Yes (1) No (2) |
|  | Ownership of owned living house | | Yes (1) No (2) |
|  | Ownership of agricultural land | | Yes (1) No (2) |
|  | Plough plow | | Yes (1) No (2) |
|  | Axe | | Yes (1) No (2) |
|  | Hoe | | Yes (1) No (2) |
|  | Shovel | | Yes (1) No (2) |
|  | Sickle | | Yes (1) No (2) |
|  | Modern beehive | | Yes (1) No (2) |
|  | Traditional beehive | | Yes (1) No (2) |
|  | Indoor plumping/ pipe water | | Yes (1) No (2) |
|  | Type of flooring | Earth/dung (0) Cement/raw wood (1) | |
|  | Toilet facility | Unsanitary or traditional pit latrine/ no toilet (0)  Sanitary or improved pit latrine (1) | |
|  | Sofa | | Yes (1) No (2) |
|  | Bed | | Yes (1) No (2) |
|  | Table | | Yes (1) No (2) |
|  | Chair | | Yes (1) No (2) |
|  | Stove | | Yes (1) No (2) |
