# Supplementary material for: High prevalence of active trachoma and associated factors among school-aged children in Southwest Ethiopia
Source: PLoS Negl Trop Dis. 2023 Dec 15;17(12):e0011846. doi: 10.1371/journal.pntd.0011846 (PMC10756553; doi:10.1371/journal.pntd.0011846)
Supplement: Attachment — Submitted filename: PNTD-D-23-00338_reviewer.pdf [file pntd.0011846.s005.pdf]

PLOS Neglected Tropical Diseases

Magnitude and factors associated with Active Trachoma among Pre-school and School-Aged children in Southwest of Ethiopia

--Manuscript Draft--

**Manuscript Number:**	PNTD-D-23-00338
**Full Title:**	Magnitude and factors associated with Active Trachoma among Pre-school and School-Aged children in Southwest of Ethiopia
**Short Title:**	Trachoma infection among school aged children
**Article Type:**	Research Article
**Keywords:**	Neglected tropical disease; magnitude; Trachoma; School age-children; Southwest Ethiopia
**Abstract:**	Introduction<br><br>Trachoma predominantly affect school-aged children, if not treated my leads to permanent blindness. This study aimed to assess the magnitude and factors associated with active trachoma among school-aged children Southwest Ethiopia, 2020.<br><br>Methods<br><br>A Community based cross-sectional study was done among 1,292 school-age children from March 1st to June 30th/2021 in Southwest Ethiopia Regional state. All school-aged children who live in this region were the source population and randomly selected school-aged children in selected kebeles during the study period were the study population. A pre-tested structured questionnaire was used to collect socio-demographic, behavioral, Environmental related information. Trachoma infections were assessed by trained health professionals following standard procedures adopted from WHO trachoma guideline. The collected data was entered using Epi data version 4.02. Descriptive analyses, bivariate and multiple variable logistic regressions were done using SPSS version 23.<br><br>Result<br><br>In this study, the Magnitude of Active Trachoma among school-aged children was 43.6% 95% CI (40.9, 46.4). age group 5-9 [AOR = 1.35 (95% CI: (1.09,2.07)]. female children [AOR = 17.13 (95% CI: (11.62, 25.23)]. House hold which do not have hand washing facility after toilet [AOR = 2.35 (95% CI: (1.66,3.34)]. wash hand after toilet [AOR = 2.23(95% CI: 1.09,2.56)]. poor sanitation status [AOR = 1.20 (95% CI: (1.02,1.57]. unimproved drinking water [AOR = 4.27 (95% CI: (2.74,6.63)]. unclean face [AOR = 2.16 (95% CI: 1.26, 3.39)]; were factors associated with active trachoma among school-aged children.<br><br>Conclusion<br><br>According to this study, environmental and behavioral variables contributed to the high prevalence of active trachoma in school-aged children. Interventions should therefore focus on improving target group behavior and surroundings.
**Additional Information:**	
**Question**	**Response**
**Government Employee**<br><br><br>Are you or any of the contributing authors an employee of the United States government?	No - No authors are employees of the U.S. government.

Powered by Editorial Manager® and ProduXion Manager® from Aries Systems Corporation

Manuscripts authored by one or more US Government employees are not copyrighted, but are licensed under a CC0 Public Domain Dedication, which allows unlimited distribution and reuse of the article for any lawful purpose. This is a legal requirement for US Government employees.<br><br>This will be typeset if the manuscript is accepted for publication.	
**Financial Disclosure**<br><br>Enter a financial disclosure statement that describes the sources of funding for the work included in this submission and the role the funder(s) played. This includes grants and any commercial funding of the work or authors.<br><br>This statement will be typeset if the manuscript is accepted for publication.<br><br>Review the submission guidelines and the instructions link below for detailed requirements and guidance.	Mizan-Tepi University provided funding for this study. The University issued the research topic and a request for proposals. From the proposal through the finalization of the research, the university research directorate supervised and evaluated it.
**Competing Interests**<br><br>On behalf of all authors, disclose any competing interests that could be perceived to bias this work.<br><br>This statement will be typeset if the manuscript is accepted for publication.<br><br>Review the instructions link below and PLOS NTDs' competing interests policy to determine what information must be disclosed at submission.	No
**Data Availability**	The data set can be accessible from the corresponding author up on reasonable request.

Provide a **Data Availability Statement** in the box below. This statement should detail where the data used in this submission can be accessed. This statement will be typeset if the manuscript is accepted for publication.<br><br>Before publication, authors are required to make all data underlying their findings fully available, without restriction. Review our PLOS Data Policy page for detailed information on this policy. Instructions for writing your Data Availability statement can be accessed via the Instructions link below.	

Powered by Editorial Manager® and ProduXion Manager® from Aries Systems Corporation

manuscript Click here to access/download;Manuscript;manuscript.docx

Magnitude and factors associated with Active Trachoma among Pre-school and School-Aged children in Southwest of Ethiopia

Dawit Getachew [1]*, Gizachew Ayele[2], Eyob Tekalgn[3], Teshale Worku[4], Abyot Asres[5], Mengistu Ayenew[6,] Fekede Woldekidan[7], Yordanos Bekele[8], Samrawit Sleshi[9]

[156789]Department of Public Health, School of Public Health, College of Medical Health Sciences, Mizan Tepi University

[234]Department of Microbiology, College of Medical Health Sciences, Mizan Tepi University

[*]Corresponding author:

Email: getdawit2011@gmail.com

Abstract

Introduction: Trachoma predominantly affect school-aged children, if not treated my leads to permanent blindness. This study aimed to assess the magnitude and factors associated with active trachoma among school-aged children Southwest Ethiopia, 2020.

Methods: A Community based cross-sectional study was done among 1,292 school-age children from March 1st to June 30th/2021 in Southwest Ethiopia Regional state. All school-aged children who live in this region were the source population and randomly selected school-aged children in selected kebeles during the study period were the study population. A pre-tested structured questionnaire was used to collect socio-demographic, behavioral, Environmental related information. Trachoma infections were assessed by trained health professionals following standard procedures adopted from WHO trachoma guideline. The collected data was entered using Epi data version 4.02. Descriptive analyses, bivariate and multiple variable logistic regressions were done using SPSS version 23.

Result: In this study, the Magnitude of Active Trachoma among school-aged children was 43.6% 95% CI (40.9, 46.4). age group 5-9 [AOR = 1.35 (95 % CI: (1.09,2.07)]. female children [AOR = 17.13 (95 % CI: (11.62, 25.23)]. House hold which do not have hand washing facility after toilet [AOR = 2.35 (95 % CI: (1.66,3.34)]. wash hand after toilet [AOR = 2.23(95% CI: 1.09,2.56)]. poor sanitation status [AOR = 1.20 (95 % CI: (1.02,1.57]. unimproved drinking water [AOR = 4.27 (95 % CI: (2.74,6.63)]. unclean face [AOR = 2.16 (95 % CI: 1.26, 3.39)]; were factors associated with active trachoma among school-aged children.

Conclusion: According to this study, environmental and behavioral variables contributed to the high prevalence of active trachoma in school-aged children. Interventions should therefore focus on improving target group behavior and surroundings.

Key Word: Neglected tropical disease, magnitude, Trachoma, School age-children, Southwest Ethiopia

Background

Trachoma which is the primary infectious cause of blindness in the world caused by the bacteria called Chlamydia trachomatis. In endemic communities Trachoma spread by eye-seeking flies, fomites and direct finger-eye contact with an infected person's discharge from their eyes or nose, as well as by direct human contact (through hands, clothing, or bedding) [1-7]. Trachoma is also among Neglected tropical disease that affects poorest communities of the world; that affects a billion people worldwide each year, their effect include Long-term disability, barriers to work, education, and economic empowerment, and a higher dependency rate stigma [8-12].

School aged children are most vulnerable to infections of this NTDs, 75% of all disability-adjusted life years from communicable diseases globally occur in children under 14 years of age [3].

In Ethiopia government has identified and focused on eight priority NTDs for control and elimination. Trachoma were among five NTDS for which there is large-scale preventive chemotherapy [13]. The government of Ethiopia worked with cross-sectorial government initiatives, NTD donors and implementing partners to join together the consortium of partners required to bring about massive scale-up of the SAFE strategy to achieve Sustainable Development Goals (SDGs) under the 2030 Agenda which directly or indirectly related with eliminations of NTDS like trachoma [14, 15].

Ethiopia has the highest prevalence of active trachoma in sub-Saharan Africa with average prevalence of around 40 %, accounting for 30% of the estimated trachoma disease burden. In Ethiopia, prevalence of blindness and low vision in the country is 1.6% and 3.7%, respectively which is highest in sub-Saharan Africa [2, 11, 16-21]. According to the national trachoma mapping of Ethiopia revealed that 568 woredas, or 68% of those in the country, have trachomatous inflammation-follicular (TF) infection of above 10% prevalence. The total population requiring MDA intervention in these woredas is more than 70 million people, the largest need of any country in the world[22].

increase in the distance to a water point, no or poor utilization of liquid waste disposal in the child’s household increase chance of Trachoma infection [2, 21].

Methods and Materials

Study Area and Study Period

This study was done in Southwest people regional state of Ethiopia, from March 1st to June 30th/2021. This region is home for more than 13 ethnic group, living in urban semi urban and pastoral structure. The region is administratively divided in to six zones; namely, Kaffa zone, Bench Sheko Zone, Sheka zone, West Omo Zone, Dawro zone and Konta zone. The total population of the region is around 3.5 million. In this area, there are 134 health centers and 836 health post two zonal hospital,10 primary level hospitals one teaching and referral hospital.

Study design

Community based Analytical cross- sectional study was done

Source and Study population

All school aged children who live in in Bench-Shako, Kaffa, Sheka and West-Omo Zone, were source population of the study. All Randomly selected school aged children in selected kebeles during the study period were the study population

Inclusion and exclusion criteria

All school-aged children living in the district for at least 6 months was included in the study. While, children who are severely ill and unable to respond or participate in the study, Children whose age is less than 5 years where excluded from the study

Sample size & sampling procedures

The sample size was calculated for both objective using Open epi software by considering the following assumptions: $Z_{\alpha/2}$ at 95% confidence interval, margin of error (d) 5%.prevalence of trachoma infection among school aged children 17.5 %, design

effect of 2. And For the second objective, ODF status variable used from previous study OR 2.52 [23], the final sample size was 1320.

Sampling technique

From southwest regional state, three zones were randomly selected by lottery method, from each zone, then from these zones, three Woreda were randomly selected again from each selected Woreda four kebeles was selected by using lottery method. Then total number school aged children from each selected kebeles during study period was identified from family folder and/or information from health extension worker and sampling frame was generated. Based on total number of school aged children identified, the required sample proportionally allocated to each selected kebeles and finally simple random sampling technique was employed to recruit study participant into study if more than one school-aged child lived in the same household, one child was selected randomly using the lottery method. When mother-child pairs were not available at the time of the data collection, two repeated visits was made, and when the same situation occurred the next household was considered for the survey.

Variables of the study

Dependent variable

Active Trachoma Infection

Independent variable

Demographic characteristics of the study participants: Age and sex of the child, religion, educational status of care taker.

Behavioral factors: face and hand washing practices, Trimming nail periodically, eating unwashed or uncooked vegetable or fruits, not washing hands after defecation and not deworming children regularly, swimming in the lake and children who were involved in to irrigation activities

Environmental: fece aroud the household, irrigational activity around household, open-field defecation, liquid waste disposal

WASH related factors: primary source of water, amount of water consumed per day, distance to water source and availability of latrine facilities, defecation site at home

Operational Definitions

School-aged children: all children between the ages of **6** and **15** years, regardless of whether they attend school [19, 24] .

Improved drinking water sources: The household that have the potential to deliver safe water by nature of their design and construction, those that are likely to be protected from outside contamination, and from faecal matter in particular. Improved water sources include household connections, public standpipes, boreholes, protected dug wells, protected springs and rainwater collection [25-27].

Unimproved water sources include unprotected wells, unprotected springs, surface water (e.g. river, dam or lake), vendor-provided water, bottled water (unless water for other uses is available from an improved source) and tanker truck-provided water[25-27].

Improved sanitation facilities are those designed to hygienically separate excreta from human contact. Improved sanitation includes flush or pour-flush to piped sewer system, septic tank pit latrines, ventilated-improved pit latrines, or pit latrines with slab or composting toilets [25, 27].

Shared or public-use sanitation facilities are not considered to be improved. Also, flush or pour-flush to elsewhere, pit latrines without slabs or open pits, bucket latrines, hanging latrines or open defecation are not considered to be improved sanitation[25, 27].

TF is defined as the presence of five or more follicles of greater than 0.5 mm in diameter in the central part of upper tarsal conjunctiva.

TI is pronounced inflammatory thickening of the tarsal conjunctiva that obscures more than half of the normal deep tarsal vessels.

Active trachoma was defined as the presence of TF or TI according to the WHO Guidelines [14, 28].

Clean face: Absence of an ocular or nasal discharge on the face of child at the time of the visit.

Clean finger: Absence of dirty materials on fingers and fingers' nail.

Data collection tools procedure, personnel and Data quality assurance

Data were collected using a structured and pretested questionnaire, observational checklist and and physical diagnosis of the eye [29-36]. The questionnaires include socio demographic, environmental and behavioral factors. The parents (mothers or head of the household) were interviewed to collect information on socio-demographic characteristics the household (sex of HH head, education status of HH head, occupation of HH head, family size and number children aged 1 to 9 years, marital status, religion, and ethnicity.), and source of drinking water, ownership of domestic animals, amount of water consumed per day, distance to water source and availability of latrine facilities which was collected from caregivers. Sex of child, age of child, frequency of face washing and using soap for face washing were also assessed by questioning a HH resident. Furthermore, time taken to fetch water, amount of water for domestic consumption, ownership of cattle and utilization of latrine were assessed by questioning a HH resident. The volume of water consumed per day was calculated by estimating the volume of fetched water per day and dividing it by the number of family members. A round trip distance to water source measured in hour. In addition, data for water sanitation and hygiene practice was collected from caregivers.

Observation check list: Variables assessed through observation by the research team included: number of rooms, separate room for cattle and cooking, presence of window in the cooking room, availability of latrine, type of latrine and distance of latrine from living house. Solid waste system availability, liquid waste disposal, presence of hand washing facility and feces seen in the compound were also assessed through observation by the research team. Moreover, cleanliness of child face and fingers, ocular discharge and nasal discharge were also assessed through observation by the research team.

Clinical diagnosis for trachoma

Clinical eye examination for the presence or absence of active trachoma was performed for each child using a simple system for the assessment of trachoma and its complications [37]. Four public health professionals who are capable of eye examination for trachoma and in clinical diagnosis of active trachoma among the study participants were recruted.

Simultaneously each child's face was examined for facial cleanliness by the health officers and the presence or absence of ocular/nasal discharge properly recorded.

Binocular loupes manufactured by Donegan optical company, Inc. at USA (×2.5 magnifications) and penlight torches was used during eye examinations. Each eye was examined separately by using binocular examination loupes lenses (× 2.5). The examination of the eye was done by careful inspection of eye lashes, cornea, limbus, eversion of the upper lid and inspection of the tarsal conjunctiva. The right eyes followed by the left examined to avoid failure to recall in which eye the examiner saw an abnormality. Then, the diagnosed children classified according to WHO simplified trachoma-grading card as trachomatous inflammation-follicular (TF), trachomatous inflammation-intense (TI), and trachomatous scarring (TS) or free from trachoma [38]. All trachoma-positive individuals was treated with topical tetracycline eye ointment and advised to consult the nearby health center for further eye health follow-ups. The principal investigator and the supervisors closely monitored the entire data collection processes. The filled-out questionnaires and eye examination results collected and delivered to supervisors after checking for consistency and completeness on daily bases. The presence of active trachoma was assessed by TF and TI.

Data processing and analysis

Data were coded and then entered, cleaned and edited using Epi data version 4.02 then exported into SPSS version 20.0 software for processing and analysis. A basic descriptive analysis was done to characterize independent factors. Crude odd ratios and AOR was computed to assess the presence and degree of association between the variables. Both bivariate and Multiple logistic regression was fitted to assess the association between outcome and explanatory variables. Those independent variables that are significant in bivariate logistic regression at 0.25 was entered in to the multivariate logistic regressions model. Backward stepwise regression used for choosing determinant variables. Extent of strength was be presented using odds ratios and its 95% confidence intervals. P-value less than 0.05 was used as a cutoff point to determine statistical significance in multiple logistic regressions the final model. Hosmer and Lemeshow as well as omnibus test were

used to test the model fitness. Multicollineaity was checked by using standard error and VIF factor. Finally, the result presented by using texts, tables, charts and graphs.

Ethical Consideration

Ethical clearance and approval was obtained from the Ethical and research Committee of Mizan-Tepi University. Further permission was obtained from zonal health department. Confidentiality was maintained by making the data collectors aware not to record any identification information. Written consent for study participation was sought from each household member present at the time of interview: adult consent, parental consent, and assent from children aged 7–16 years. For have active trachoma infection tetracycline was provided and appropriate referral linkage was made.

Result

Socio-demographic characteristics of the study participants

The response rate of this study was 93.23%, the median age of the school aged children participated in this study is 11 with inter quartile range 3. Only 6.1% of participants were from urban and also 82.1% the study participant were from 1st cycle primary school (Table 1).

Table 1 Socio-demographic characteristics of study participant (n = 1292)

Variables	Category	Frequency
Child age	6-10	639(51)
	11-15	612(49)
Child sex	Male	610(47)
	Female	682(53)
Child educational level cat(cycle)	First cycle (1-4)	890(82)
	Second cycle(5-8)	196(18)
Educational enrollment status	Enrolled	746(58)
	Not enrolled	367(28)
	Dropout	179(14)
Residence	Rural	1137(88)
	Urban	155(12)
Maternal educational status	No formal education	51(3.9)
	Read and write	131(10.1)
	Primery1-8	819(63.4)
	Secondary and above	291(22.5)
Maternal occupational status	House wife	831(64)
	Private employee	34(3)
	Government employee	201(16)
	Merchant	226(17)
Wealth index	Poor	609(47)
	Medium	420(33)
	Rich	263(20)

Household, Environmental and Behavioral characteristics

Regarding the Environmental and behavioral factors distribution and Sources of drinking water, majority of the household do not have toilet and those who have toilet lacks hand washing after toilet (fig 1and fig 2)

The prevalence of Trachoma infection among School Aged children in Southwest Ethiopia

The prevalence of trachoma among school aged children in the study area was 43.6% 95% CI (40.9, 46.4) (fig 3)

Factor associated with trachoma among School Aged Children

In this study the odds of trachoma infection was 1.35 times higher among children in age group between 5-9 as compared to school aged children in age group 10-15 [Adjusted OR = 1.35 (95 % CI: (1.09,2.07)]. Also, the odds of trachoma infection was 17 times higher among female school aged children than male children [Adjusted OR = 17.13 (95 % CI: (11.62, 25.23)]. Also, the odds of trachoma infection was 2.35 times higher among school aged children who were from house hold which do not have hand washing facility after toilet [Adjusted OR = 2.35 (95 % CI: (1.66,3.34)]. And the odds of trachoma infection was 2.23 times higher among those children who wash their hand after toilet [AOR = 2.23(95% CI: 1.09,2.56)]. The odds of trachoma infection was 1.20 times higher among school aged children exposed to poor sanitation status [Adjusted OR = 1.20 (95 % CI: ((1.02,1.57)]. Also unimproved drinking water increased the odds of trachoma infection 4.27 times as compared to school aged children from household who have improved drinking water [Adjusted OR = 4.27 (95 % CI: ((2.74,6.63)]. In addition, the odds of trachoma infection was 2.16 times higher among school aged children whose face was unclean [Adjusted OR = 2.16 (95 % CI: 1.26, 3.39)].

Table 2 Bivariate and Multivariate analysis of factors associated with trachoma infection among School aged children in Southwest Ethiopia, 2020

	Trachoma infected	Not infected	COR(95%CI)	AOR(95% CI)
**Age of the school aged children**				
6-10	253	427	1.81(1.45,2.85)*	1.35(1.09,2.07)**
11-15	317	295	1	1
**Gender**				
Male	435	146	1	1
Female	135	576	12.71(9.75,16.57)*	17.13(11.62,25.23)**
**Grade**				
First cycle	252	293	1	
Second cycle	242	352	1.25(.98,1.58)	
**Maternal educational status**				
literate	130	161	1	
Illiterate	440	561	1.03(0.79,1.86)	
**Mother occupational status**				
Paid work	133	165	1	
Unpaid work	437	557	1.02(.79,1.33)	
**Washing hand after latrine**				
Yes	352	489	1	1
No	494	616	2.65(2.07, 3.38)	2.23(1.09,2.56)**
**Hand washing facility**				
Yes	330	430	1	1
No	315	376	1.13(.93,1.40)	1.20(.87,1.81)
**Sanitation**				
Improved	252	339	1	1
Unimproved	417	506	1.22(.95, 1.57)*	1.20(1.01,1.57)**
**Drinking water source**				
Unimproved	137	203	3.54(2.74, 4.57	4.27(2.74,6.63)**
Improved	296	325	1	1
**Child face**				
Clean	258	388	1	1
Not clean	333	601	1.37(1.09,1.71)*	2.16(1.26,3.69)**

Discussion

This study revealed that the magnitude of active Trachoma infection was 44.1% with 95% CI (40.9, 46.4) in the study area. This distribution very high when compared to similar studies result in Ethiopia [23, 28, 39-44], and abroad [45, 46]. This variation may be due to the fact that this study attempted to reach school-aged children from remote communities where access to health care is not optimal and that it also included students who were not enrolled in school, who are often not beneficiaries of school-based interventions. However, the prevalence of active trachoma in this study is relatively lower than report from Ethiopia [16, 47] and abroad In addition to this the current study included school aged children aged from 6-16 but most trachoma survey is done among children aged 1-9 year Moreover, this finding implies the less or the non-implementation of failed of Trachoma control and prevention strategies

Regarding the factors associated with active Trachoma infection among School aged children, the result of multiple logistic regression analysis showed that children in age group between 5-10, female children, children who were from house hold which have hand washing facility after toilet, improved sanitation and water source were significantly associated with trachoma infection

In this study, the odds of trachoma infection were 1.35 times higher among children in age group between 5-9 as compared to school aged children in age group 10-15. Younger children are more risk group for trachoma infection, due to lack of hand washing practice. This finding is in line whith finding from similar research. Also, the odd of trachoma infection was 17 times higher among female school aged children than male children.

The odds of trachoma infection was 2.35 times higher among school aged children who were from house hold which have hand washing facility after toilet. Moreover, The odds of trachoma infection was 2times higher among school aged children who have unclean face [23]. This is in line with similar research finding presence of nasal and ocular

discharge attracts flies, which transmit the bacteria from infected children to health children.

Finally, the odds of trachoma infection were 1.20 times higher among school aged children exposed to poor sanitation status. This, result is in line with finding from similar research. Dirty environment is favorable site of insect breeding. Also unimproved drinking water increased the risk of trachoma infection 4.27 times as compared to school aged children from household who have improved drinking water. This shows shortage of clean water or lack of proper utilization of water in household is the reason for poor personal hygiene to household member including children to keep their hand and face clean

Limitations of the study

The limitation of this study is that the study participant were all school aged children but WHO recommend survey of trachoma infection on children aged 1-9, however most intervention are school based therefore assessing school aged children both enrolled and not enrolled children is important.

Conclusion and Recommendation

This study, the Magnitude of trachoma among School-aged children in the study area was 44% 95% CI (40.9, 46.4). Regarding the factors associated with Trachoma, the study has found children in the age group between 5-10, children who were from households that have hand washing facilities after toilet were significantly associated with trachoma infection in the study area. To control and eliminate this high prevalence of trachoma the local government and partners should focus on the socio demographic factors and behavioral factor to prioritize those at highest risk, and also planner and implementer shall consider the improvement of behavioral and environmental intervention like hand washing at critical time, facial cleanness and health education intervention.

Abbreviation

AOR: Adjusted Odds Ratio, CI : Confidence Interval, HBM: Health Believe Model: SWE:South West Ethiopia, TPB: Theory of planned behavior, WHO: World Health Organization

Declarations

Ethics approval and consent to participate

This study was carried out in accordance with the Helsinki Declaration and with the approval of Mizan Tepi University's Research ethics committee with Ethics approval. Each Zone and health office department once again provided a letter of cooperation. Before beginning the study, all participants provided written informed consent after discussing the aims, purpose, and risks and benefits of the study. Furthermore, throughout the study, confidentiality, anonymity, and the freedom to withdraw from the study at any time were respected.

Consent for publication

Not applicable

Availability of data and materials

The data set can be accessible from the corresponding author up on reasonable request.

Competing interests

The authors declare that they have no competing interests.

Funding

Mizan-Tepi University provided funding for this study. The University issued the research topic and a request for proposals. From the proposal through the finalization of the research, the university research directorate supervised and evaluated it.

Authors' contributions

DG and FW wrote the proposal, participated in data collection, analyzed the data, and drafted the paper. All other author together with DG and FW approved the proposal with some revisions, participated in data analysis, and revised subsequent drafts of the paper. All authors commented and approved the final manuscript.

Acknowledgments

We would like to acknowledge Mizan-Tepi University for Covering the cost of the project and monitoring the conduct of the study. our data collectors and study participant of this study.

Reference

1. Enriquez, M., *Vaccine Hesitancy and COVID-19: Nursing's Role.* Hisp Health Care Int, 2021. 19(2): p. 74.
2. Ayanaw Tsega Ferede, A.F.D., Amare Tariku and Akilew Awoke Adane, *Prevalence and determinants of active trachoma among preschool-aged children in Dembia District, Northwest Ethiopia.* BioMed Central Ltd, 2017. 6(128).
3. Dr Lorenzo Savioli Director, D.o.C.o.N.T.D.W.H.O.G., Switzerland, *Neglected tropical diseases, hidden successes, emerging opportunities,* D.o.C.o.N.T.D.N.W.H. Organization, Editor. 2009, World Health Organization: Geneva 27, Switzerland.
4. Peeling, R.W., D.I. Boeras, and J. Nkengasong, *Re-imagining the future of diagnosis of Neglected Tropical Diseases.* Comput Struct Biotechnol J. 15: p. 271-274.
5. Azzarone, B., et al., *Pathogenic Mechanisms of Vaccine-Induced Immune Thrombotic Thrombocytopenia in People Receiving Anti-COVID-19 Adenoviral-Based Vaccines: A Proposal.* Front Immunol, 2021. 12: p. 728513.
6. Hollingsworth, T.D., *Counting Down the 2020 Goals for 9 Neglected Tropical Diseases: What Have We Learned From Quantitative Analysis and Transmission Modeling?* Clin Infect Dis. 66(suppl_4): p. S237-S244.
7. Koukounari et al, *Integrated monitoring and evaluation and environmental risk factors for urogenital schistosomiasis and active trachoma in Burkina Faso before preventative chemotherapy using sentinel sites.* BMC Infect Dis, 2011. 11(191).
8. WHO, *Neglected Tropical Disease*. 2018, WHO: Geneva
9. Hotez PJ, A.M., Basa´n˜ez M-G, Bolliger I, Bourne R, et al., *The Global Burden of Disease Study 2010: Interpretation and Implications for the Neglected Tropical Diseases.* PLOS Neglected Tropical Diseases, 2014. Volume 8 (Issue 7).
10. Dr Lorenzo Savioli Director, D.o.C.o.N.T.D.W.H.O.G., Switzerland. *Neglected Tropical Diseases* 2018 11/30/2018 [cited 2018 30/11/2018]; Available from: http://www.who.int/neglected_diseases/director/en/.
11. Hotez PJ, S.L., Fenwick A, *Neglected Tropical Diseases of the Middle East and North Africa: Review of Their Prevalence, Distribution, and Opportunities for Control.* PLOS Neglected Tropical Diseases, 2012. 6(2).

12. Dr Matshidiso Moeti. *The Expanded Special Project for Elimination of Neglected Tropical Diseases*. 2018 30/11/2018; Available from: http://espen.afro.who.int/system/files/content/resources/ESPEN_Institutional%20Brochure_For_An_Africa_Free_Of_NTDs.pdf.
13. Mengitsu, B., et al., *Ethiopia and its steps to mobilize resources to achieve 2020 elimination and control goals for neglected tropical diseases: Spider webs joined can tie a lion.* International Health, 2016. 8(suppl_1): p. i34-i52.
14. *WHO Alliance for the Global Elimination of Blinding Trachoma by the year 2020. Progress report on elimination of trachoma, 2013.* Wkly Epidemiol Rec, 2014. 89(39): p. 421-8.
15. Bangert, M., et al., *The cross-cutting contribution of the end of neglected tropical diseases to the sustainable development goals.* Infect Dis Poverty. 6(1): p. 73.
16. Berhane, Y., et al., *Prevalence and causes of blindness and low vision in Ethiopia.* Ethiopian Journal of Health Development, 2007.
17. Peter J. Hotez, A.K., *Neglected Tropical Diseases in Sub-Saharan Africa: Review of Their Prevalence, Distribution, and Disease Burden.* PLOS Neglected Tropical Diseases, 2009. 3(8).
18. Health, F.D.R.o.E.M.o., *Second Edition of National Neglected Tropical Diseases Master Plan 2015/16 – 2019/20 (2008-2012 EFY)*, M.o. Health, Editor. 2016, Ministry of Health: Addis Ababa.
19. Nebiyu Negussu et al, *Ethiopia Schistosomiasis and Soil-Transmitted helminths control programme; Progress, and prospect*, f.m.o. health, Editor. 2017, ministry of health: Addis Ababa.
20. Berhane, Y., et al., *Prevalence of Trachoma in Ethiopia.* Ethiopian Journal of Health Development, 2008.
21. Emerson PM, N.J., Biru E, Graves PM, Ejigsemahu Y, et al, *Integrating an NTD with One of ''The Big Three'': Combined Malaria and Trachoma Survey in Amhara Region of Ethiopia.* PLoS Negl Trop Dis, 2008. 2(3).
22. Belete Mengitsua et al, *Ethiopia and its steps to mobilize resources to achieve 2020 elimination and control goals for neglected tropical diseases: Spider webs joined can tie a lion.* International Health, 2016. 5(240).
23. Delelegn, D., et al., *Status of active trachoma infection among school children who live in villages of open field defecation: a comparative cross-sectional study.* BMC Public Health, 2021. 21(1): p. 2051.
24. Organization, W.H., *Soil-transmitted helminthiases: eliminating as public health problem soil-transmitted helminthiases in children: progress report 2001-2010 and strategic plan 2011-2020.* 2012.
25. WHO, *Global nutrition monitoring framework: operational guidance for tracking progress in meeting targets for 2025*. 20 December 2017: Geneva.
26. *The drinking water ladder*.
27. Centers for Disease Control and Prevention, N.C.f.E.a.Z.I.D.N., *Assessing Access to Water & Sanitation*, W. Foodborne, and Environmental Diseases at CDC, Editor. 2017.
28. Asres, M., M. Endeshaw, and M. Yeshambaw, *Prevalence and Risk Factors of Active Trachoma among Children in Gondar Zuria District North Gondar, Ethiopia.* Journal of Preventive Medicine, 2016. 01(01).

29. Tefera, E., et al., *Prevalence and intensity of soil transmitted helminths among school children of Mendera Elementary School, Jimma, Southwest Ethiopia.* Pan Afr Med J, 2017. 27: p. 88.
30. King, J.D., et al., *Intestinal Parasite Prevalence in an Area of Ethiopia after Implementing the SAFE Strategy, Enhanced Outreach Services, and Health Extension Program.* PLOS Neglected Tropical Diseases, 2013. 7(6): p. e2223.
31. Ganguly, S., et al., *High prevalence of soil-transmitted helminth infections among primary school children, Uttar Pradesh, India, 2015.* Infect Dis Poverty, 2017. 6(1): p. 139.
32. Freeman, M.C., et al., *Associations between school- and household-level water, sanitation and hygiene conditions and soil-transmitted helminth infection among Kenyan school children.* Parasites & Vectors, 2015. 8(1): p. 412.
33. Alelign, T., A. Degarege, and B. Erko, *Soil-Transmitted Helminth Infections and Associated Risk Factors among Schoolchildren in Durbete Town, Northwestern Ethiopia.* Journal of Parasitology Research, 2015. 2015: p. 641602.
34. Kattula, D., et al., *Prevalence & risk factors for soil transmitted helminth infection among school children in south India.* Indian Journal of Medical Research, 2014. 139(1): p. 76-82.
35. Sady, H., et al., *Prevalence and Associated Factors of Schistosomiasis among Children in Yemen: Implications for an Effective Control Programme.* PLOS Neglected Tropical Diseases, 2013. 7(8): p. e2377.
36. WoldeKidan, E., et al., *Prevalence of active trachoma and associated factors among children aged 1 to 9 years in rural communities of Lemo district, southern Ethiopia: community based cross sectional study.* BMC Infectious Diseases, 2019. 19(1): p. 886.
37. Thylefors, B., et al., *A simple system for the assessment of trachoma and its complications.* Bulletin of the World Health Organization, 1987. 65(4): p. 477-483.
38. WHO, *simplified trachoma grading system. Community Eye Health*. 2004, PMC free article: Geneva p. 68
39. Basha, G.W., A.A. Woya, and A.K. Tekile, *Prevalence and risk factors of active trachoma among primary school children of Amhara Region, Northwest Ethiopia.* Indian Journal of Ophthalmology, 2020. 68(5): p. 750-754.
40. Sata, E., et al., *Twelve-Year Longitudinal Trends in Trachoma Prevalence among Children Aged 1-9 Years in Amhara, Ethiopia, 2007-2019.* Am J Trop Med Hyg, 2021. 104(4): p. 1278-1289.
41. Lemma, E. *Prevalence and Risk Factors of Trachoma among Children of Woreillu W oreda , South W 0110 Administrative Zone*. 2014.
42. Ayelgn, K., T. Guadu, and A. Getachew, *Low prevalence of active trachoma and associated factors among children aged 1–9 years in rural communities of Metema District, Northwest Ethiopia: a community based cross-sectional study.* Italian Journal of Pediatrics, 2021. 47(1): p. 114.
43. Tadesse, B., et al., *The burden of and risk factors for active trachoma in the North and South Wollo Zones of Amhara Region, Ethiopia: a cross-sectional study.* Infectious Diseases of Poverty, 2017. 6(1): p. 143.
44. Melkie, G., M. Azage, and G. Gedamu, *Prevalence and associated factors of active trachoma among children aged 1-9 years old in mass drug administration*

graduated and non-graduated districts in Northwest Amhara region, Ethiopia: A comparative cross-sectional study. PLOS ONE, 2020. 15(12): p. e0243863.

45. Favacho, J., et al., *Prevalence of trachoma in school children in the Marajó Archipelago, Brazilian Amazon, and the impact of the introduction of educational and preventive measures on the disease over eight years.* PLOS Neglected Tropical Diseases, 2018. 12(2): p. e0006282.

46. Xue, W., et al., *A Cross-Sectional Population-Based Survey of Trachoma among Migrant School Aged Children in Shanghai, China.* BioMed Research International, 2016. 2016: p. 8692685.

47. Golovaty, I., et al., *Access to Water Source, Latrine Facilities and Other Risk Factors of Active Trachoma in Ankober, Ethiopia.* PLOS ONE, 2009. 4(8): p. e6702.

Supporting information

Figure 1 Environmental and behavioral factors distribution among School aged children in southwest Ethiopia, 2020

Figure 2 Sources of drinking water in Southwest Ethiopia, 2020

Figure 3 prevalence of Trachoma infection among School Aged children in Southwest Ethiopia

Click here to access/download;Figure;S1 fig 1.docx

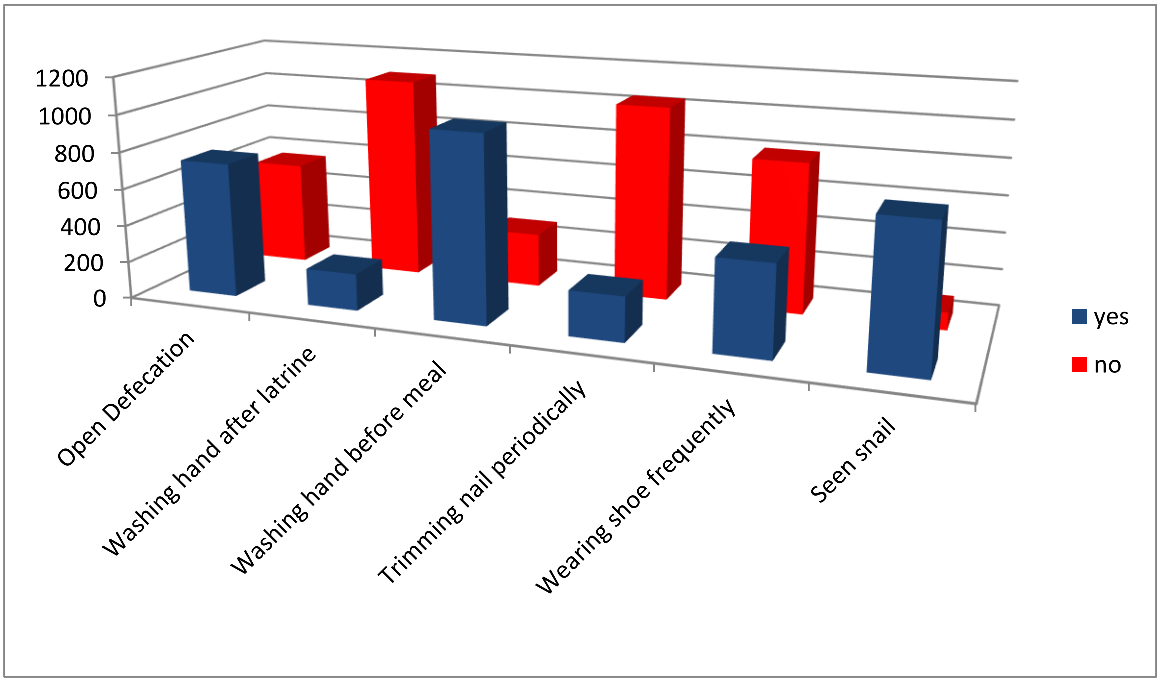

Figure 1 Environmental and behavioral factors distribution among School aged children in

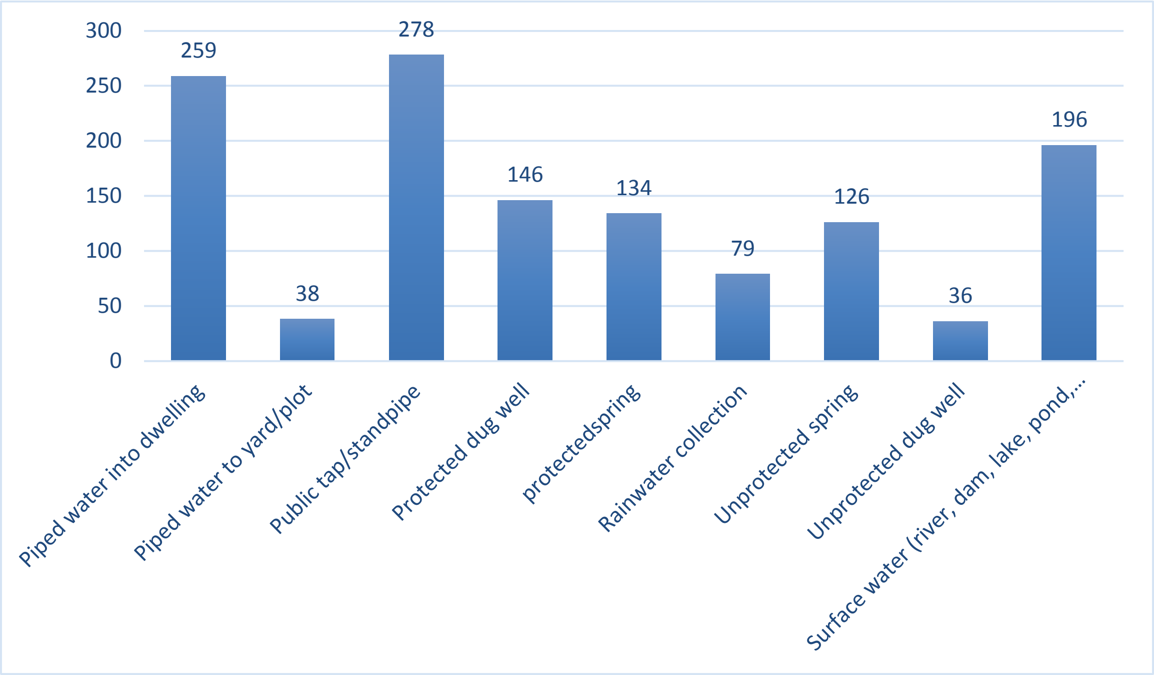

Figure 1 Sources of drinking water in Southwest Ethiopia, 2020

Click here to access/download;Figure;S3 fig 3.docx

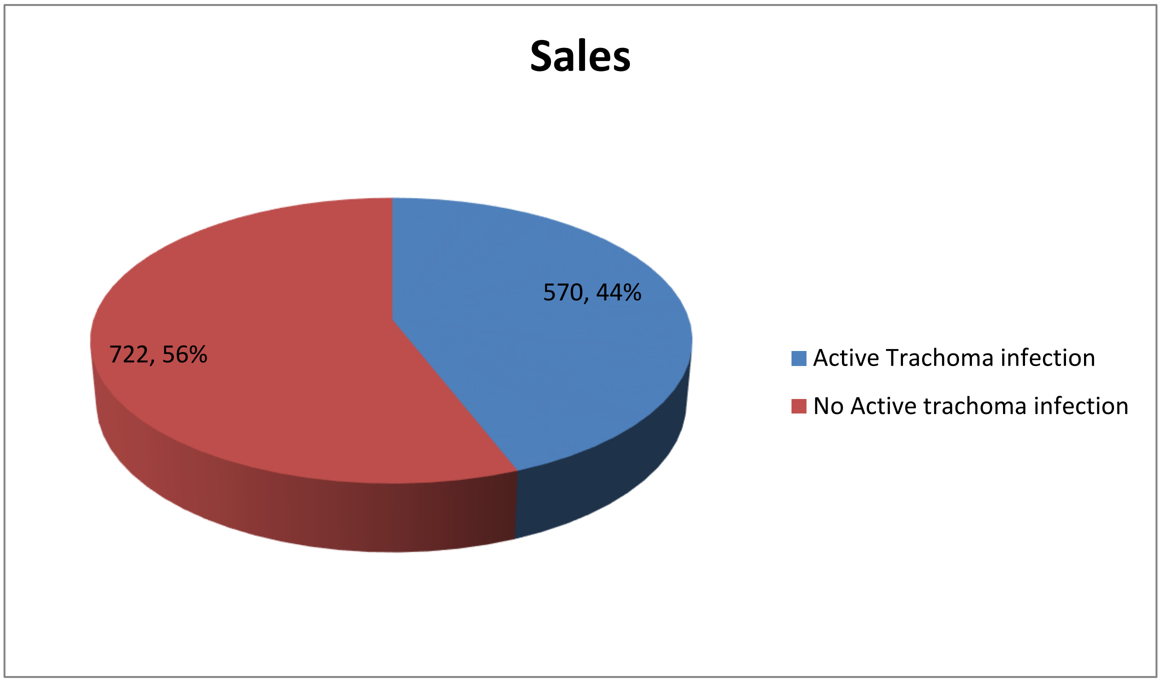

Figure 1 prevalence of Trachoma infection among School Aged children in Southwest Ethiopia

Questionnaire

Click here to access/download

Supporting Information

trachoma tool.docx
