# Supplementary material for: High prevalence of active trachoma and associated factors among school-aged children in Southwest Ethiopia
Source: PLoS Negl Trop Dis. 2023 Dec 15;17(12):e0011846. doi: 10.1371/journal.pntd.0011846 (PMC10756553; doi:10.1371/journal.pntd.0011846)
Supplement: Attachment — Submitted filename: PLOS Neglected Tropical Diseases.docx [file pntd.0011846.s004.docx]

PLOS Neglected Tropical Diseases

**Magnitude and factors associated with Active Trachoma among Pre-school and**

**School-Aged children in Southwest of Ethiopia:**

Dear Editor,

Thanks for giving me the opportunity to review this paper. The paper addresses an import topic for sub-saharan Africa where Trachoma is endemic yet can be controlled. The Authors have tried to bring out the factors associated with active trachoma among school-aged children. The factors they present are very well know fact the only addition is that this information comes from a new area where such information has not been collected. I do believe they would make this study strong if they looked at the factors of affecting the trachoma elimination in the area as they have alluded to in line 294.

The objective is not well stated in the article apart from the abstract and there is lot of grammar that need to be corrected to make some statements clear to the reader. The use of abbreviation is common and not expounded e.g., HH could mean hand hygiene.

I would recommend the authors to relook at their data analysis and presentation to really make a n impact of the data collected. They seem to be mixing those with higher odds of getting the trachoma.

Authors need to also make the reader have a clear picture of the area of study, by explaining the various division of the areas to general acceptable divisions like towns or villages or clusters. For example, its hand to understand what Woreda and Kebeles means. Are they equivalent of a village or a town or division/district.

In the discussion authors also need to bring out the factors they found as associated with trachoma how it’s different with other published work elsewhere or similar and how does that help with interventions being put in place in Ethiopia.

**Abstract:**

1. “if not treated my leads to permanent blindness.” Correct the sentence. I think the authors met **may** instead of **my**
2. Not clear what the author means by **“House hold which do not have hand washing facility after toilet**” and difference between this and “wash hand after toilet” these could be almost the same. since one cannot wash hands if the facilities are not available but am cognizance that facilities may be available, and people miss washing the hands. I think most import issue to bring out is those with hand washing facilities and subjects didn’t wash hands.
3. Line 55: grammar
4. Line 78: First time in use please write in full not all understand MDA “**population requiring MDA intervention**”
5. The study design is not very clear how the children were selected apart from the author mentioning that they were randomly selected. Please clarify what you mean by Woreda and Kebeles for the reader to be able to understand your sampling frame.
6. Line 120 -123 are just hanging not making sense, may forgot to indicate the dependent and independent variables.
7. Line 128: not deworming children regularly I don’t think its fits as a behavioral factor.
8. Line 189, spelling of “recruited”.
9. Who was data collected maintained, although no identification information was collected, how was the data stored to avoid loss or land into other hands.?
10. Line 247: private employee, doesn’t mean self-employed or employed in a private institution?
11. Spelling: Primary
12. Was data on the age of the household head collected? There is association of education, age and cleanness.
13. Line 266-268: And the odds of trachoma 267 infection was 2.23 times higher among those children who wash their hand after toilet 268 [AOR = 2.23(95% CI: 1.09,2.56)]. This contradicts line 273-274.
14. Line 286-287 and 291-291 seems to contradict each other.
15. Line 294-295 talk of failed strategy yet the authors talk of poor access to health care line 289.
16. Line 303: young children are known to get behaviors better than older children. I think it would be better to talk of guardians here than children since this state cant be generalized for younger children and hand washing.
17. Line 304: Reference for the study
18. Line 306: check the data in the table and the description.
19. On limitation of the study the authors are not clear how the WHO recommendation affects their study and what mitigation was put in place to such limitation.
20. Apart from the abstract the objective of the study is not clear on the article especially under introduction. This needs to come out clear on the body of the article.
