# Supplementary material for: High prevalence of active trachoma and associated factors among school-aged children in Southwest Ethiopia
Source: PLoS Negl Trop Dis. 2023 Dec 15;17(12):e0011846. doi: 10.1371/journal.pntd.0011846 (PMC10756553; doi:10.1371/journal.pntd.0011846)
Supplement: Attachment — Submitted filename: Response to Reviewers.docx [file pntd.0011846.s006.docx]

**Reviewer's comments Questions**

1. **Comment 1 by Reviewer #1:** Dear Editor, Thanks for giving me the opportunity to review this paper. The paper addresses an import topic for sub-saharan Africa where Trachoma is endemic yet can be controlled. The Authors have tried to bring out the factors associated with active trachoma among school-aged children. The factors they present are very well know fact the only addition is that this information comes from a new area where such information has not been collected. I do believe they would make this study strong if they looked at the factors of affecting the trachoma elimination in the area as they have alluded to in line 294.

**Author response:** Thank you for your constructive comment, as you have stated several studies have been done in Ethiopia regarding the prevalence and factor associated with active Trachoma, However, beside the geographical variation, almost all of these studies were done on children aged 1-9. The current study evaluated the prevalence of trachoma and associated factor among school-aged children (6-16), new population besides the new area. Moreover, the current studies encourage local health authority to conduct regular survey on the prevalence of trachoma.

2. **Comment 2 by Reviewer #1:** The objective is not well stated in the article apart from the abstract and there is lot of grammar that need to be corrected to make some statements clear to the reader.

**Author response:** Thank you dear reviewer I have added, the objective on main body of the manuscript. ***‘The objective of this study was to assess the prevalence and factors associated with active trachoma among school-aged children in Southwest Ethiopian Peoples Regional State.’*** **Page number 4, line number 89-91.**

3. **Comment 3 by Reviewer #1:** The use of abbreviation is common and not expounded e.g., HH could mean hand hygiene.

**Author response:** Thank you dear reviewer, we have corrected the usage of common abbreviation; HH is to mean Household. We have introduced each abbreviation in full form in their first appearance with the abbreviation in bracket. Also, we have provided the list of abbreviation. ***‘AOR: Adjusted Odds Ratio, CI : Confidence Interval, ITI: International Trachoma Initiative, COR: Crud Odds Ratio: HEW: Health Extension Worker, MDA: Mass Drug Administration, NTD: Neglected Tropical Diseases, SAC: School-Age Children, SAFE: Surgery, Antibiotics, Face cleanliness and Environmental sanitation, SNNPR: Southern Nations, Nationalities and Peoples Region, SWEPRS: Southwest Ethiopia Peoples Regional State, TF : Trachomatous Inflammation – Follicular, TI: Trachomatous Inflammation – Intense, TS: Trachomatous Conjunctival Scarring, TT : Trachomatous Trichiasis, WASH: Water, Sanitation and Hygiene, WHA: World Health Assembly, WHO: World Health Organization.’* Page number 17, line number 311-319.**

4. **Comment 4 by Reviewer #1:** I would recommend the authors to relook at their data analysis and presentation to really make an impact of the data collected. They seem to be mixing those with higher odds of getting the trachoma.

**Author response: *Thank you dear reviewer, we have relooked our data analysis and presentation, we have reanalysed the data by including variables which we had omitted in the analysis, this is because the research originally evaluated selected NTDS (STH, Schistosomiasis, and Trachoma) in the study area, this cause large number of variables which lead the analysis to be incomplete. Doing this have improved the revised manuscript greatly.***

5. **Comment 5 by Reviewer #1:** Authors need to also make the reader have a clear picture of the area of study, by explaining the various division of the areas to general acceptable divisions like towns or villages or clusters. For example, its hand to understand what Woreda and Kebeles means. Are they equivalent of a village or a town or division/district?

**Author response:** Thank you dear reviewer, in the revised manuscript we have made a clear description of the study area. This makes clear how the region is administratively subdivided. The term Woreda was equivalent with districts and kebele was the lowest administrative division in Ethiopia. ***‘******This study was done in Southwest Ethiopia Peoples Regional State (SWEPRS) from March 1st to June 30th, 2021. This region is a newly established that was split off from SNNPR. The region is constituted by six zones: Kaffa, Bench-Sheko, Sheka, West-Omo, Dawro, and Konta. In this zone there are 41 districts and 16 city administrations. In these districts and city administrations, there are 903 kebeles (a kebele is the lowest legal administrative division in Ethiopia). Total population of the region is 3.3 million.’ Page number5.line number 94-99***

6. **Comment 6 by Reviewer #1:** In the discussion authors also need to bring out the factors they found as associated with trachoma how it’s different with other published work elsewhere or similar and how does that help with interventions being put in place in Ethiopia.

**Author response:** Thank you dear reviewer, we have accepted the comment, now in the discussion section we have elaborated how the factors associated with trachoma in the study area and, how this finding is different from similar research finding in different part of Ethiopia.

**Comments by Reviewer #2:**

1. **Comment 6 by Reviewer #2:** Design of the study is good; however, lot of extraneous data has been collected which does not form a part of the analysis and is not discussed and hence irrelevant and also unethical. There is no novelty value and hence the significance, unless it can be shown in the discussion of this study, how it is different from previous ones carried out in Ethiopia on this subject. The general execution has been satisfactorily rigorous but the scholarship leaves a lot to be desired.

**Author response:** Dear Reviewer, Thank you for your comment, the extraneous variable collected here is not only for to assess factors associated with trachoma, initially this study is part of study assessing factors associated with selected neglected tropical disease (NTDs), the study area , the NTDs assessed were (STH, Schistosomiasis and trachoma), due to this the large data set lead, the author to include extraneous variable mistakenly included the analysis and previous manuscript. However, we have a different tool for trachoma assessment we have included in additional file. Therefore, the data were re analysed and presented, in the edited manuscript as per the comment by removing non relevant and considering previously left variable.

**Question by Reviewer #2**

1. **Question 1 by Reviewer #1:** Objectives not clearly stated.

**Response for reviewer 1**: thank you dear reviewer, we have accepted the comment and we have included the objective clearly in the revised manuscript **‘The objective of this study was to assess the prevalence and factors associated with active trachoma among school-aged children in Southwest Ethiopian Peoples Regional State.’ Page number 4, line number 89-91.**

2. **Question 2 by Reviewer #1:** The sampling procedures not clear. e.g., from the study region it's difficult to know the number of kebeles, to have a picture that they were representative.

**Response for reviewer 1**: Thank you dear reviewer, we have accepted the comment and we have added information on the number of kebele in the study area. ***‘This study was done in Southwest Ethiopia Peoples Regional State (SWEPRS) from March 1st to June 30th, 2021. This region is a newly established that was split off from SNNPR. The region is constituted by six zones: Kaffa, Bench-Sheko, Sheka, West-Omo, Dawro, and Konta. In this zone there are 41 districts and 16 city administrations. In these districts and city administrations, there are 903 kebeles (a kebele is the lowest legal administrative division in Ethiopia). Total population of the region is 3.3 million.’ Page number5.line number 94-99***

Thus the sampling technique can be more clear, ***‘A multi-stage sampling technique was used to reach the study subject. From the six zones in SWEPRS, three zones, (Bench-Sheko, Kaffa, and West-OmO) were randomly selected by the lottery method. From these three zones, 30% districts and city administrations were selected by stratification. From each selected strata representative kebeles selected by using the lottery method. Based on the estimated number of school-aged children in each kebele, the required sample was proportionally allocated to each selected kebele. A sampling frame was generated and a simple random sampling technique was employed to recruit study participants*.’ Page number 6, line number 124-131**

3. **Question 3 by Reviewer #1:** Looks like the authors used multi-stage sampling and would be nice to say from zones how the Woredas an kebeles were selected because the zone I do believe don't have equal Woredas/Kebeles. this would also enable proper description of study population and ensure bias is eliminated through stratification. the sample size is difficult to say weather is adequate because of limited information.

**Response for reviewer 1**: Thank you dear reviewer, we have accepted the comment and we have added in formation on the sample size and the sampling procedure and how we proportionally selected number of kebele in the to keep representativeness. ***‘A multi-stage sampling technique was used to reach the study subject. From the six zones in SWEPRS, three zones, (Bench-Sheko, Kaffa, and West-OmO) were randomly selected by the lottery method. From these three zones, 30% districts and city administrations were selected by stratification. From each selected strata representative kebeles selected by using the lottery method. Based on the estimated number of school-aged children in each kebele, the required sample was proportionally allocated to each selected kebele. A sampling frame was generated and a simple random sampling technique was employed to recruit study participants.’ Page number 6, line number 124-131***

4. **Question 4 by Reviewer #1:** confidentiality of the data, being safe is not well stated.

**Response for reviewer 1**: Thank you dear reviewer, we have accepted the comment and we have corrected the statement regarding confidentiality of the data ***‘Furthermore, throughout the study, confidentiality, anonymity, and the freedom to withdraw from the study at any time were respected. ‘Confidentiality was maintained by making the data collectors aware not to record any identification information. The data was kept safe under strict supervision by the principal investigator. The participants were told that the interview may take a maximum of 30–45 minutes. ‘Page number9, line number 196-199***

5. **Question 5 by Reviewer** #1: the limitations not well articulated. the authors poorly discuss how the data can be helpful to advance our understanding of the topic. the topic is very relevant in public health, but little is addressed.

**Author response:** Thank you dear reviewer for your comment, we agree we your comment. In the revised manuscript we have articulated the limitation of the study. Also we have addressed how the finding is helpful to advance the knowledge of the concerned body to implement, monitor and evaluate the elimination strategies of Trachoma in the study area. ‘***Besides, the study design was cross-sectional, which is difficult to show temporal relation of the variables social desirability bias can be the limitation of this study. However, the study used observational data collection technique to minimize social desirability bias. Also, this study addressed the prevalence of trachoma among school-aged children living in the newly established region of Ethiopia, where WASH status of the community was suboptimal. Thus, the finding can serve as a baseline for next intervention plan and to evaluate the progress of the 2030 trachoma elimination goal, in the study area.’ Page number 16, line number 295-301.***

6. **Question 6 by Reviewer** **#1:** Line 266-268: And the odds of trachoma 267 infection were 2.23 times higher among those children who wash their hand after toilet 268 [AOR = 2.23(95% CI: 1.09,2.56)]. This contradicts line 273-274. Authors to relook at the data to ensure its not contradicting what the data says.

**Author response:** Thank you dear reviewer for your comment, the contradiction solved in the revised manuscript. Because after relooking the data, some extraneous and variables were removed from the analysis. Thus, the discussion was rewritten by comparing the finding with previous similar studies.
**Reviewer #2**

1. **Question and comment 1 by Reviewer #2**: The objectives of the study are mentioned and the study design is appropriate. However, the term prevalence would be more appropriate than magnitude in the objective.

**Response for reviewer 2**: Thank you dear reviewer, we have accepted the comment on the title and we have replaced the term magnitude by prevalence in the title and also on the objective to be consistent. The title is ‘***High prevalence of active trachoma and associated factors among school-aged children in Southwest Ethiopia’ page number 1, line number 3***

**Question and comment 2 by Reviewer #2:** The title includes preschool children whereas the study was on school children only. This needs correction. The study population is described and appropriate. Sample size is adequate.

**Response for reviewer 2**: Thank you dear reviewer, we have accepted the comment, the research focus was on School-aged children. The title was corrected as follow. ***‘High prevalence of active trachoma and associated factors among school-aged children in Southwest Ethiopia’ page number 1, line number 3***

2. **Question and comment 3 by Reviewer #2:** Ethical considerations should include informed written consent (not just written consent) and should mention the amount of time permitted from administering the consent by researchers to provision of consent by the participant.

**Response for reviewer 3**: Thank you dear reviewer, we have accepted the comment, on Ethical considerations we have added the term informed written consent. However, during the data collecting we did not limit the time to get the consent the data collector waits until the participant understands the consent form and finish their question and provide their consent to participate. ‘***Ethical clearance and approval were obtained from the Ethics and Research Committee of Mizan-Tepi University. Moreover, permission was obtained from the zonal health department. Before beginning the study, informed written consent was sought from parents and assent from children aged 7–16 years. All participants were informed and discussed the aims, purpose, risks, and benefits of the study. Furthermore, throughout the study, confidentiality, anonymity, and the freedom to withdraw from the study at any time were respected. The data was kept safe under strict supervision by the principal investigator.* Page number 9, line number 191-197**

3. **Question and comment 4 by Reviewer #2:** The entire manuscript needs copy editing for language and grammar for better understanding

**Author response 2**: thank you dear reviewer, we admit that the entire manuscript needs copy editing, we have tried to improve the language quality in the revised manuscript.

4. **Question and comment 5 by Reviewer** #2: There have been data collected which has not been included in the analysis in the study for example socio-demographics such as marital state, income, educational status, etc, and even other observational data such as cleanliness of nails etc. All this data is irrelevant as it is not a part of the analysis.

**Author response:** thank you dear reviewer, there were some extraneous variables this is because the study was part of a study which assessed magnitude of selected NTD, in the study area, thus we have omitted some irrelevant variable with Trachoma.

5. **Question and comment 6 by Reviewer #2**: The results are not clear. The figures stated in the table do not match the figures in the manuscript, for example line 306 and 307 in the manuscript says that the the odds for trachoma was 2.35 times higher among children from houses having hand washing facility, whereas in table 2 the odds ratio is 1.2 for those children from houses wait NO handwashing facility. Please reconcile. Similarly, the statement in Discussion line 298,299 and 300 is contradictory to the results in table 2.

**Author response:** Thank you, dear reviewer, we have accepted the comment. In the revised manuscript, we have presented the result clearly and also the discussion was rewritten.

6. **Question and comment 7 by Reviewer #2**: Conclusions are contrary to the data presented as mentioned above.

**Author response:** Thank you, dear reviewer, We have made the conclusion of the study based on the result in the revised manuscript.’ ***In this study, the prevalence of active trachoma among school-aged children was 44.1 %, 95% CI (40.9- 46.41) indicating the intensive scale-up of the SAFE strategy in the study area. Also, being in age group 6–9, being female, improved water source, improved sanitation, flies seen at HH, ocular discharge, Flies on child face and the condition of the child’s face were significantly associated factors with active trachoma.* Page Number 16-17, line number 302-308**

7. **Question and comment 8 by Reviewer #2**: Recommendations are vague and not specific.

**Author response:** Thank you, dear reviewer, we have written the recommendation based on the finding in the revised manuscript. ***Thus, the local government and partners acting on improving the health of the community should focus on intensive scaling up of the SAFE strategy by giving priority to children in the age group 6-10 and female children. Also, increasing HH access improved sanitation and improved water sources help reduce the high prevalence of active trachoma. Moreover, keeping cleanness of school-age children face can help to reduce trachoma prevalence in the study area.* Page Number 16-17, line number 302-3012**

8. **Question and comment 9 by Reviewer #2**: (Reference no 2) What would be interesting however is to discuss the reasons for the significant difference in prevalence in both these studies which has not been done. Other than this difference and its explanation this study has not furthered our understanding of trachoma and its determinants.

**Author response:** Thank you, dear reviewer, for your comment, But the reference number 2 (***Ayanaw Tsega Ferede, A.F.D., Amare Tariku and Akilew Awoke Adane, Prevalence and determinants of active trachoma among preschool-aged children in Dembia District, Northwest Ethiopia. BioMed Central Ltd, 2017. 6(128)),*** cited on previous manuscript was done on pre-school aged children (children age less than 5), but the current study was done on school aged children (children aged 6-16), In addition to this, there is a 1500km geographical distance between, these two-studies study area. Thus, it is difficult to generalize the previous study for the current study area. Moreover, the previous study assessed only a single district, the current study assed 12 district of Southwest Ethiopians people’s region. Due to endemicity of trachoma, this region requires attention of the local and global communities acting on the improvement of the life and health of the impoverished communities.

9. **Question and comment 8 by Reviewer #2**: The entire manuscript needs a good copy editing for better accuracy brevity and clarity.

**Author response:** Thank you dear reviewer we have accepted the comment and we have copy edited the manuscript. Now the revised manuscript is clearer and more understandable.

10. **Question and comment 9 by Reviewer #2**: The figures need proper titles. The pie chart for prevalence of trachoma has a title 'Sales'.

**Author response:** Thank you dear reviewer, regarding the figure labelling we have corrected on the revised manuscript. We apologies for the mistake.

**Reviewer #3:**

1. **Question and comment 1 by Reviewer #3:** The language is confusing and hard to follow.
   Proofreading is mandatory.

**Response for reviewer 3**: thank you dear reviewer, we admit that the entire manuscript needs copy editing, we have tried to improve the language quality in the revised manuscript.

2. **Question and comment 2 by Reviewer #3:** Validation of the used questionnaire in trachoma?

**Response for reviewer 3**: thank you dear reviewer, we have used a pretested and validated questionnaire, the tools was used on different studies in Ethiopia. ***The questionnaire was pretested on 5% of the final sample size in none selected, but similar area and population with the actual study setup.***’ **Page number 9, Line number 178-179**

3. **Question and comment 3 by Reviewer #3:** Was it used in the native language?

**Author response: T**hank you dear reviewer, The interview questionnaire was prepared in English, but translated in to Amharic which is the working language of the region.

4. **Question and comment 4 by Reviewer #3**: Review tables and figure labels and numbers
   the word "SALES" in the third fig?

**Author response:** Thank you dear reviewer, we apologies for the error we meant, we have corrected the lable and title for tables and figures. The word "SALES", a mistake on the excel it should be Trachoma infection status, we have corrected it on the revised manuscript.

5. **Question and comment 5 Reviewer #3:** in the discussion section, it would be catching to the readers' eyes to summarize the reports [23, 28, 39-44], [45, 46] in a table.
   **Author response:** Thank you dear reviewer, we have made clear the discussion on the prevalence of trachoma by comparing with previous studies. However, making the summarize with table would be more appropriate if the study was systematic review and meta analysis,
