# Supplementary material for: High prevalence of active trachoma and associated factors among school-aged children in Southwest Ethiopia
Source: PLoS Negl Trop Dis. 2023 Dec 15;17(12):e0011846. doi: 10.1371/journal.pntd.0011846 (PMC10756553; doi:10.1371/journal.pntd.0011846)
Supplement: Attachment — Submitted filename: response to Reviewer.docx [file pntd.0011846.s007.docx]

**Reviewer's Responses to Questions**


**Reviewer #2:** Issues mentioned in the previous review have been addressed in the revised manuscript. However it will be ethical to mention in the manuscript, that the data collected in this study is a part of a larger database which was collected to assess the magnitude of  NTDs in the region

**Author response:** Dear reviewer thank you for your comment, in the revised manuscript we have mentioned that this manuscript was based on the data collected to assess the magnitude of NTDs in the SWERPS.’’ **A Community based Analytical cross- sectional study was done in SWEPRS from the 1st of March to the 30th of June, 2021, as part of a broader research theme ‘prevalence of selected neglected tropical diseases among school-aged children in SWEPRS.’** Page number 4, line number 68-70.
Reviewer #2: Issues mentioned in the previous review have largely  been addressed. However there are some glaring errors. For example the conclusion states that improved water source and improved sanitation is associated with increased prevalence of active trachoma, whereas in the same conclusion it is stated that presence of animal feces near the HH is associated with increased active trachoma. there is need to use appropriate language or else the meaning conveyed is exactly the opposite.

**Author response:** Dear reviewer thank you for your comment, we have corrected inappropriately articulated sentence in the revised manuscript to keep the message conveyed is logical and based on the study resalt.

***‘This study found that the prevalence of active trachoma among school-aged children was very high when compared with the WHO threshold. Also, age group 6–10, female gender, presence of ocular and nasal discharge, unclean face of the child’s, presence of flies on the child's face, and at HH have significantly associated with increased odds of active trachoma among school-aged children, while access to improved water sources and improved sanitation lowered the odds of active trachoma among school-aged children. Thus, to reduce the high prevalence of active trachoma in the study area, intensifying and scaling up the facial cleanness and environmental and sanitation components of the SAFE strategy is needed by giving great attention to those in the age group 6–10 and female gender.’’*** Page number 15, line number 251-259

Reviewer #2: Conclusions need to be rewritten keeping in mind the meaning to be conveyed. The likely reasons for the gender disparity need to explained better. Limitations need to be rewritten for better clarity of the points raised.

**Author response:** Dear reviewer thank you for your comment, in the revised manuscript we have rewritten to the conclusion. Page number 15, line number 251-259

We have also tried to explain the gender disparity based on the study result comparing with available literature.

***‘The gender disparity in acquiring active trachoma among girls can be associated with the gender role that baby girls play in taking care of younger family members who are potential sources of trachoma infection. This disproportionate risk can persist even in the later age of the girl as a woman because of the raising of children, which increases the recurrent acquisition of the infection, complications, and blindness.’’ Page 13, line number 223-227***

Moreover, we have made the limitation of the study clearer.

***The potential limitation of this study was cross-sectional nature of the design, which is difficult to show temporal relation of the variables. Also, social desirability bias can be another limitation of this study. But addressing most trachoma impacted population, with relatively large sample size and large geographical coverage is the strength of the study. ’’*** Page 14, line number 246-249.

Reviewer #2: The whole manuscript needs a proper copy editing for language and grammar. For example: in the introduction part of the abstract, the second sentence begins with a 'Which'. There are spelling mistakes such as bing for being etc,

**Author response:** Dear reviewer thank you for your comment, we have copy edited the whole manuscript for English language quality and grammar issue.

Reviewer #2: The issues raised in the previous review have been addressed satisfactorily, except for the language and grammar editing.

**Author response:** Dear reviewer thank you for your comment, we have copy edited the whole manuscript for English language quality and grammar issue.

Reviewer #3: The authors did well in the revision, however few concerns are raised.
1- Several typos and grammatical errors, for example, the 2nd sentence in the conclusion (same one in the authors summary doc), the word "cheek list", "Inanition"....etc.
2- Regarding the validation, the authors' response is not identical the the text cited. Also, this is not the ideal validation. What was the result of this pilot testing; not mentioned in the results.
**Author response:** Dear reviewer thank you for your comment, regarding the validation of the tool used to collect the study variable; the tool was pretested on 5% of the sample.

Figure Files:
While revising your submission, please upload your figure files to the Preflight Analysis and Conversion Engine (PACE) digital diagnostic tool, [https://pacev2.apexcovantage.com](https://pacev2.apexcovantage.com/). PACE helps ensure that figures meet PLOS requirements. To use PACE, you must first register as a user. Then, login and navigate to the UPLOAD tab, where you will find detailed instructions on how to use the tool. If you encounter any issues or have any questions when using PACE, please email us at [figures@plos.org](mailto:figures@plos.org).

**Author response:** Thank you dear editor, we have used PACE to ensure figure meet PLOS requirements.

Data Requirements:
Please note that, as a condition of publication, PLOS' data policy requires that you make available all data used to draw the conclusions outlined in your manuscript. Data must be deposited in an appropriate repository, included within the body of the manuscript, or uploaded as supporting information. This includes all numerical values that were used to generate graphs, histograms etc.. For an example see here: <http://www.plosbiology.org/article/info%3Adoi%2F10.1371%2Fjournal.pbio.1001908#s5>.

**Author response:** Thank you dear editor, we have made all the data used to draw conclusion of this manuscript available within the manuscript and uploaded as supporting information.
Reproducibility:
To enhance the reproducibility of your results, we recommend that you deposit your laboratory protocols in [protocols.io](http://protocols.io/), where a protocol can be assigned its own identifier (DOI) such that it can be cited independently in the future. Additionally, PLOS ONE offers an option to publish peer-reviewed clinical study protocols. Read more information on sharing protocols at <https://plos.org/protocols?utm_medium=editorial-email&utm_source=authorletters&utm_campaign=protocols>

**Author response:** Thank you dear editor, however the current manuscript was based on the World Health Organization Clinical trachoma grading guideline.
References
Please review your reference list to ensure that it is complete and correct. If you have cited papers that have been retracted, please include the rationale for doing so in the manuscript text, or remove these references and replace them with relevant current references. Any changes to the reference list should be mentioned in the rebuttal letter that accompanies your revised manuscript. If you need to cite a retracted article, indicate the article's retracted status in the References list and also include a citation and full reference for the retraction notice.

**Author response:** Thank you dear editor, we have reviewed our reference and removed those outdated and included relevant reference. We have indicated removed reference and newly added relevant in the track changed.
